# The Concentration of Selected Inflammatory Cytokines (IL-6, IL-8, CXCL5, IL-33) and Damage-Associated Molecular Patterns (HMGB-1, HSP-70) Released in an Early Response to Distal Forearm Fracture and the Performed Closed Reduction With Kirschner Wire Fixation in Children

**DOI:** 10.3389/fendo.2021.749667

**Published:** 2021-12-09

**Authors:** Marzena Tylicka, Tomasz Guszczyn, Michał Maksimowicz, Joanna Kamińska, Ewa Matuszczak, Maria Karpińska, Olga Martyna Koper-Lenkiewicz

**Affiliations:** ^1^ Department of Biophysics, Medical University of Białystok, Białystok, Poland; ^2^ Department of Pediatric Orthopaedics and Traumatology, Medical University of Białystok, Białystok, Poland; ^3^ Department of Clinical Laboratory Diagnostics, Medical University of Białystok, Białystok, Poland; ^4^ Department of Pediatric Surgery and Urology, Medical University of Białystok, Białystok, Poland

**Keywords:** high mobility group protein B1 (HMGB1), heat shock protein 70 (HSP70), interleukin 6 (IL-6), interleukin 8 (IL-8), interleukin 33 (IL-33), chemokine CXCL5, distal forearm fracture, closed reduction with percutaneous Kirschner wire fixation (CRKF)

## Abstract

The evaluation of trauma after surgery through objective analysis of biochemical markers can help in selecting the most appropriate therapy. Thus the aim of the study was the evaluation of the concentration of selected inflammatory cytokines (IL-6, IL-8, CXCL5, IL-33), C-reactive protein (CRP), and damaged-associated molecular patterns (DAMPs): HMGB-1, HSP-70 in the plasma of children in response to bone fracture and 12-14 hours after subsequent surgery performed by closed reduction with percutaneous Kirschner wire fixation (CRKF). The study will answer the question if the CRFK procedure leads to excessive production of inflammatory and damage markers. Blood samples from 29 children with distal forearm fractures were collected 30 min. before CRKF procedure and 12-14 hours after performance of the procedure. The control group was composed of 17 healthy children. IL-6 and CRP concentrations were analyzed using routinely performed *in vitro* diagnostics tests; the remaining proteins were analyzed with the use of the ELISA method. Increased values of IL-6, CRP, and HSP-70 represented an early inflammatory response to distal forearm fractures classified as SH-II type according to the Salter-Harris classification system. However, the median CRP concentration was within the reference values not indicative of inflammation. The CRKF procedure may be a good solution for the treatment of bone fractures, as damaged associated molecular patterns – HMGB-1 and HSP-70 – did not significantly differ 12-14 hours after the approach was applied as compared to the control group. Moreover, the increase in IL-6 concentration after the CRKF procedure was 1.5-fold to the level before CRKF, while the increase of this marker in response to the distal forearm fracture was 4.3-fold compared to the control group. Based on this data, it appears reasonable to suggest that the CRKF approach caused less damage and inflammatory response in comparison to the response to the fracture itself.

## Introduction

Distal forearm fractures caused by falls around the home or during sports activities are the most common injuries in pediatric traumatology (1). There is no standard treatment for pediatric distal forearm fractures. They can be treated surgically or non-operatively. The treatment method depends on the degree of angulation, the patient’s age, the type of fracture, and the surgeon’s preference. Most of these fractures, especially in children under 10 years of age and angulation less than 10-20 degrees, are treated non-operatively by closed reduction and cast immobilization. In contrast, significantly displaced and unstable fractures should be treated surgically by Close Reduction and Internal Fixation (CRIF). Kirschner wires (K-wires) or titanium elastic nails (TEN) can be used for internal bone stabilization. For displaced, unstable distal forearm fractures **C**lose **R**eduction and the distal radius percutaneous **K**irschner wire **F**ixation (CRKF) have been advocated as a safe and reliable technique for maintaining the alignment of the fracture and avoiding re-displacement and further manipulation (2). Lieber et al. (3) suggest a trans epiphyseal intramedullary K-wire fixation in unstable diametaphyseal forearm fractures as a quick, minimally invasive, and technically easy treatment option.

Bone fracture trauma causes systemic inflammation with leukocytosis and the release of cytokines within 24 hours after the occurrence of the incident (4). Inflammation is the protective response of tissue leading to the initiation of the healing process (5). Just after injury, neutrophils are the first to arrive at the fracture site and are one of the most important cells in the post-fracture inflammatory response. The recruited neutrophils express and produce chemokines (e.g. CXCL5, IL-8/CXCL8), which participate in the healing process by attracting immune cells (6). Bone fracture also promotes the release of damage-associated molecular patterns (DAMPS), such as HMGB-1 or heat shock protein 70 (HSP-70), and alarmin cytokines such as interleukin-33 (IL-33). These DAMPS, together with IL-33, play an important role in the orchestration of tissue homeostasis, including repair and remodeling processes (7, 8).

Due to improved medical technology, public health education, changes in lifestyle, minimal hospitalization requirements, and parents’ expectations, there is a current trend toward pediatric surgical treatment to obtain perfect outcomes while minimizing the high rates of overtreatment (9, 10). For these reasons, the evaluation of traumatization through objective analysis of biochemical markers can help in selecting the most appropriate therapy.

Moreover, eventual healing is highly dependent on the inflammatory phase, and the deficiency or inhibition of inflammation can lead to impaired bone healing. Also, an excessive inflammation increases the risk of impairing the healing process. Therefore, an analysis of the inflammatory markers may be valuable in the prediction of the course of regeneration processes (5).

Thus the aim of the study was the evaluation of the concentration of selected inflammatory cytokines (IL-6, IL-8, CXCL5, IL-33), C-reactive protein (CRP), and DAMPs (HMGB-1, HSP-70) in the plasma of children in response to bone fracture and investigation into how the closed reduction with percutaneous Kirschner wire fixation (CRKF) affected the levels of these molecules 12-14 hours after the surgical procedure. The conducted study will answer the question if the CRFK procedure leads to excessive production of inflammatory and damage markers. Such comprehensive analysis has not been performed thus far.

## Material and Methods

### Patients

The study population comprised 29 skeletally immature children admitted between 2019-2020 to the Pediatric Orthopaedics and Traumatology Department of the Medical University of Bialystok due to distal forearm fracture, directed to CRKF procedure. **Table 1** presents demographical data and clinical characteristics of children with distal forearm fractures (**Table 1**).

**Table 1:** Demographical data and clinical characteristics of children with distal forearm fracture.

Patients	Children with distal forearm fracture directed for CRKF procedure (N=29)
**Gender**	Female N=12 (41%)
	Male N=17 (59%)
**Age**	5-15 y. (median, 10 y.)
**Weight**	20-58 kg (median, 32 kg)
**Bone fracture etiology**	Fall N=27 (93%)
	Traffic accident N=2 (7%)
**CRKF duration**	20-60 minutes
	up to 20 min. N=15 (52%)
	above 20 min. N=14 (48%)
**Type of anesthesia**	short intravenous anesthesia
**Intraoperative/postoperative complications**	none
**Length of hospitalization**	1-2 days*
	1 day N=27 (93%)
	2 days N=2 (7%)

N, number of cases; y, years; kg, kilograms; CRKF, closed reduction with percutaneous Kirschner wire fixation.

*CRKF procedure does not require longer hospitalization than 2 days.

All fractures resulted from falls from low-energy trauma, followed by injury at the playground or contact activities. The inclusion criteria were: distal forearm displace fractures involving the physis, skeletally immature children, female or male patients aged between 5-15, the CRKF lasting up to 60 min., and no comorbidities. Fracture displacement in the current study was defined when the angulation of the fracture was greater than 45° or when the fracture had less than 50% of bony contact or when there was a complete displacement of the fracture in the initial injury. In all patients, fractures were classified according to the Salter-Harris classification system as SH-II type (11). Exclusion criteria were: hospital admission later than 3 hours after trauma, preexisting infections, or diseases that required long-term medication.

Those children whose parents gave informed consent for both clinical and biochemical follow-up were included in the study.

17 healthy children, aged-matched to the study group, whose parents gave informed consent for both clinical and biochemical follow-up, served as a control group.

Approval for this study was obtained from the Local Bioethics Committee (Permission No: R-I-002/45/2018). Procedures were following the ethical standards set out in the Declaration of Helsinki put out by World Medical Association.

### Plasma IL-6, IL-33, IL-8, CXCL5, HSP70, and HMGB1 Concentration Evaluation

Blood samples from children with distal forearm fracture were collected into test tubes with EDTA-K (S-Monovette^®^ 1.2 ml K3E, SARSTEDT) 30 min., before closed reduction with percutaneous Kirschner wire fixation (pre-CRKF) and 12-14 hours after performance of the procedure (post-CRKF). After the centrifugation (20 min. at 1000 g force) plasma samples were aliquoted and stored at -80°C until further analysis.

IL-6 concentration was measured using an *in vitro* diagnostics electrochemiluminescence immunoassay (ECLIA) test on the COBAS e411 analyzer (Catalogue No. 05109442) from Roche Diagnostics, Penzberg, Germany, according to the manufacturer’s instructions. Plasma samples were not diluted before analysis. The detection range of the assay test is 1.5-5000 pg/ml. The manufacturer of the assay kit referred to the diagnostic repeatability coefficient of variation (CV%) as 2.9% at IL-6 mean concentration of 12.1 ± 0.346 pg/mL and diagnostic precision CV% as 3.1% at IL-6 mean concentration of 12.1 ± 0.371 pg/mL.

IL-33, IL-8, CXCL5, HSP70, and HMGB1 concentrations were analyzed using the ELISA (enzyme-linked immunosorbent assay) method in compliance with the manufacturer’s instructions. Plasma samples for IL-33, IL-8, HSP70, and HMGB1 concentration evaluation were not diluted before analysis. Plasma samples for CXCL5 concentration evaluation were diluted 2-fold before analysis.

IL-33 concentration was measured using a Human IL-33 ELISA kit (Catalogue No. D3300B) from R&D Systems, Abingdon, UK. The detection range for the kit is between 3.13-200 pg/mL, the mean minimum detectable dose (sensitivity) of the assay is 0.357 pg/mL. The manufacturer of the assay kit referred to the intra-assay coefficient of variation (CV%) as 3.7% at IL-33 mean concentration of 31.0 ± 1.15 pg/mL and inter-assay CV% as 4.4% at IL-33 mean concentration of 31.0 ± 1.35 pg/mL. The average recovery of IL-33 concentration throughout the range of the assay for EDTA-K plasma is 91% (range 83-103%).

CXCL5 concentration was measured using a Human CXCL5/ENA-78 ELISA kit (Catalogue No. DX000) from R&D Systems, Abingdon, UK. The detection range for the kit is between 31.3-2000 pg/mL, the mean minimum detectable dose (sensitivity) of the assay is 15 pg/mL. The manufacturer of the assay kit referred to the intra-assay coefficient of variation (CV%) as 5.3% at CXCL5 mean concentration of 244 ± 12.9 pg/mL and inter-assay CV% as 7.4% at CXCL5 mean concentration of 240 ± 17.8 pg/mL. The average recovery of CXCL5 concentration throughout the range of the assay for EDTA-K plasma is 98% (range 92-104%).

IL-8 concentration was measured using a Human IL-8/CXCL8 ELISA kit (Catalogue No. D8000C) from R&D Systems, Abingdon, UK. The detection range for the kit is between 31.3-2000 pg/mL, the mean minimum detectable dose (sensitivity) of the assay is 3.5 pg/mL. The manufacturer of the assay kit referred to the intra-assay coefficient of variation (CV%) as 5.6% at IL-8 mean concentration of 168 ± 9.4 pg/mL and inter-assay CV% as 7.4% at IL-8 mean concentration of 196 ± 14.5 pg/mL. The average recovery of IL-8 concentration throughout the range of the assay for EDTA-K plasma is 103% (range 97-111%).

HSP70 concentration was evaluated using a Biorbyt Human heat shock proteins (HSP70) ELISA kit (Catalogue No. Orb397059) from Biorbyt Ltd., Cambridge, UK. The detection range for the kit is between 125-8000 pg/mL. The mean minimum detectable dose (sensitivity) of the assay is 60 pg/mL. HMGB1 concentration was measured using an EIAB Human HMGB1/high mobility group protein B1 ELISA kit (Catalogue No. E0399h) from EIAB Science, Wuhan, China. The detection range for the kit is between 0.156-10.00 ng/mL. The mean minimum detectable dose (sensitivity) of the assay is 0.1 ng/mL.

CRP concentration was measured by the *in vitro* latex enhanced immunoturbidimetric method using a Cobas Integra 400 plus analyzer (Catalogue No. 07876033190) from Roche Diagnostics, Penzberg, Germany, according to the manufacturer’s instructions. Plasma samples were not diluted before analysis. The manufacturer of the assay kit referred to the diagnostic repeatability coefficient of variation (CV%) as 3.4% at CRP mean concentration of 1.33 ± 0.0451 mg/L and diagnostic precision CV% as 4.4% at CRP mean concentration of 1.33 ± 0.0586 mg/L.

### Data Analysis

Statistical analysis was performed using the STATISTICA PL release 12.5 Program. All the results are presented as median with 25^th^ and 75^th^ percentiles. The data was tested for normality using the Shapiro-Wilk test. Because the tested parameters did not pass the normality test, results obtained in this experiment were analyzed by the Mann-Whitney U test or Wilcoxon matched pairs test (two-sided nature). The differences in continuous variable values between the study and control groups were assessed using the Mann-Whitney U test. To compare paired data obtained 30 min. before and 12-14 hours after closed reduction with Kirshner wire fixation in children with a distal forearm fracture, the Wilcoxon matched-pairs test was used. Correlations were studied by using Spearman’s Correlation test. Differences were considered significant with a value of p<0.05.

The sample size was calculated with an independent two-sided t-test by choosing a 95% confidence level, a margin of error of ± 5%, power of 80%, the size of the pediatric population in our region, and the number of cases performed per year.

## Results

The concentration of the analyzed cytokines, except for IL-33, and DAMPs was within the range of the assay kit in all plasma samples of children with a distal forearm fracture.

### Damage Associated Molecular Patterns (HMGB-1,HSP-70) Results

HSP-70 concentration measured 30 min. before the CRKF procedure was statistically higher in comparison to the control group. In contrast, we did not observe significant changes in HMGB-1 concentration as a consequence of distal forearm fracture. HMGB-1 and HSP-70 concentrations measured 12-14 hours after the CRKF procedure did not significantly differ as compared to the control group (**Table 2**).

**Table 2:** DAMPs, inflammatory cytokines and CRP concentration results in plasma of children with distal forearm fracture and in the control group.

	CHILDREN WITH DISTAL FOREARM FRACTURE (N = 29)		CONTROL GROUP (N = 17)	p-value*		
	Pre-CRKF (A)	Post-CRKF (B)	(C)	A vs C	B vs C	A vs B
**HMGB-1 [ng/mL]**	5.59 (1.23-7.23)	1.73 (0.00-4.62)	6.01 (2.96-8.03)	p=0.562	p=0.059	p=0.084
**HSP-70 [pg/mL]**	1550.00 (0.00-2200.00)	0.00 (0.00-1913.00)	0.00 (0.00-0.00)	***p=0.002***	p=0.121	p=0.178
**CXCL5 [pg/mL]**	420.20 (152.84-575.80)	318.50 (144.30-568.20)	477.30 (381.00-931.60)	p=0.131	p=0.094	p=0.592
**IL-8 [pg/mL]**	2.99 (2.36-4.28)	2.89 (2.50-5.38)	3.06 (2.45-3.93)	p=0.988	p=1.000	p=0.961
**IL-6 [pg/mL]**	8.57 (5.13-11.05)	12.50 (8.78-24.40)	1.98 (1.50-2.28)	***p<0.001***	***p<0.001***	***p<0.001***
**CRP [mg/L]**	0.48 (0.20-2.15)	2.97 (1.60-5.43)	0.09 (0.03-0.15)	***p<0.001***	***p<0.001***	***p<0.001***

Results are presented as median with 25%-75% percentiles.

DAMPS, damage-associated molecular patterns; N, number of cases; CRKF, closed reduction with percutaneous Kirschner wire fixation; HMGB-1, high mobility group protein B1; HSP-70, heat shock protein 70; CXCL5, C-X-C Motif Chemokine Ligand 5; IL-8, interleukin 8; IL-6, interleukin 6; CRP, C-reactive protein; Pre-CRKF- 30 minutes before CRKF; Post-CRKF-12-14 hours after CRKF.

* A bold p-value <0.05 is considered as showing a significant difference between groups (according to the Mann-Whitney U test or Wilcoxon matched-pairs test).

HMGB-1 and HSP-70 concentrations 12-14 hours after the CRKF procedure was performed were lower than those measured 30 min. before the CRKF procedure, but the obtained differences were not significant (**Table 2**).

### Cytokines (CXCL5, IL-8, IL-6, IL-33) Concentration Results

IL-33 concentration in all plasma samples taken from children with a distal forearm fracture 30 min. before the CRKF procedure, 12-14 hours after the CRKF procedure, and from the control group were below the detection limit of the assay kit.

CXCL5 concentration measured 30 min. before the CRKF procedure and measured 12-14 hours after the CRKF procedure was lower than the concentration obtained from the control group, but the differences were not significant. Chemokine concentration also did not statistically differ between samples obtained from children 30 min. before the CRKF procedure compared to samples obtained 12-14 hours after the CRKF procedure (**Table 2**).

IL-8 concentration measured 30 min. before the CRKF procedure and measured 12-14 hours after the CRKF procedure was almost the same as the concentration obtained in the control group. Moreover, IL-8 concentration did not differ between samples obtained from children 30 min. before the CRKF procedure compared to samples obtained 12-14 hours after it (**Table 2**).

**Figure 1:**
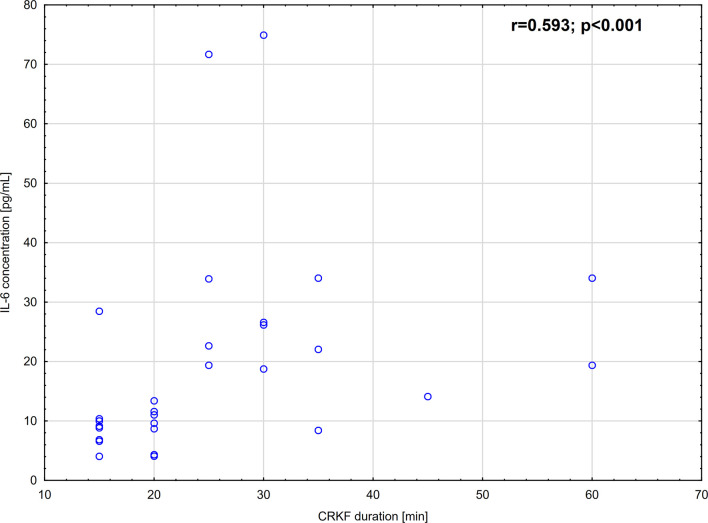
Correlation between plasma IL-6 concentration and CRKF duration.

IL-6 concentration measured 30 min. before the CRKF procedure was significantly higher than in the control group. The increase was 4.3-fold. Additionally, IL-6 concentration measured 12-14 hours after the CRKF procedure was significantly higher as compared to the control group. IL-6 concentration evaluated in samples obtained 12-14 hours after the CRKF procedure was also significantly higher as compared to samples obtained 30 min. before the CRKF procedure (**Table 2**). The increase was 1.5-fold. Moreover, we observed that IL-6 plasma concentration strongly, positively correlated with the duration of CRKF (**Figure 1**). However, we did not find any correlation between IL-6 concentration and selected DAMPs (HMGB-1, HSP-70) or C-reactive protein (CRP) concentration measured at either 30 min. before the CRKF procedure (p=0.0787; p=0.2151 and p=0.4471, respectively) or 12-14 hours after the CRKF procedure (p=0.9911; p=0.1423 and p=0.3933, respectively).

### C-Reactive Protein (CRP) Analysis

CRP concentration measured 30 min. before the CRKF procedure was significantly higher than in the control group. CRP concentration measured 12-14 hours after the CRKF procedure was also significantly higher as compared to the control group. Additionally, CRP concentration evaluated in samples obtained 12-14 hours after the CRKF procedure was significantly higher as compared to samples obtained 30 min. before the CRKF procedure (**Table 2**). However, it should be noted that in all cases the median CRP concentration was within the reference values that equal 0-10 mg/L (12).

## Discussion

The bone fracture causes an inflammatory response, which peaks 24 hours following the injury and is complete after the first week. During this time a complex cascade of pro-inflammatory signals and growth factors are released (13). Inflammation is a crucial biological process that is beneficial to fracture healing (14). Edderkaoui et al. (6) noticed that fracture induces secretion of inflammatory cytokines (IL-6, TNF-α) and chemokines (CXCL_s_), which play a role in post-fracture inflammation and fracture healing. It was further confirmed by Baht et al. (15), who postulate that during the inflammatory phase neutrophils secrete a wide range of cytokines and chemokines (IL-1, IL-6, IL-10, TNFα, CCL2, CXCL-1α, MIP-1) to attract monocytes, which are precursors of macrophages playing an integral part in bone homeostasis and bone fracture repair.

Horst et al. (16) on an animal model demonstrated that shortly after bone trauma proinflammatory mediators such as IL-6 recruit cells needed for tissue regeneration. The authors showed that serum IL-6 concentration, contrary to the IL-8 concentration, significantly increased 14 hours after trauma compared to the control group (16). A study by Volpin et al. (17) in humans showed that IL-6 and IL-8 levels could be potential biomarkers predicting the development and degree of the systemic inflammatory response in injured patients. A significantly higher concentration of these cytokines was found in severe and moderately injured groups compared to controls (17). Our results are, in general, consistent with those obtained by Horst et al. (16) and Volpin et al. (17), as we found that IL-6 concentration in children with a distal forearm fracture was significantly higher in comparison to the control group. In contrast to Volpin et al. (17), we did not observe a significant change in IL-8 concentration between the fracture and non-fracture groups. Volpin et al. suggest that cytokine concentration increases soon after injury (6 hours), while at the same time highlighting that the duration of this reaction depends on the severity of trauma (17). In this context, we have observed that the lack of IL-8 growth may be related to the fact that distal forearm fracture in our study was classified as type 2 according to Salter-Harris. This is a less serious injury and has the second least extensive anatomic involvement of physeal tissue (11). Significant growth of IL-6 concentration may suggest it is a more sensitive marker of early inflammatory response to distal forearm fracture.

In the current study, we also demonstrated significant growth of IL-6 concentration 12-14 hours after the CRKF approach in comparison to the samples collected 30 min. before surgical procedure. Similar results were obtained by Edderkaoui et al. (6), who showed an increase of IL-6 concentration immediately after surgery and re-equilibration to baseline within 24 hours post-surgery (6). We moreover demonstrated that IL-6 concentration 12-14 hours after the CRKF procedure was still significantly higher in comparison to the control group. However, it should be mentioned that the increase in IL-6 concentration compared to the level before CRKF was 1.5-fold, while the increase of this marker in response to the distal forearm fracture was 4.3-fold compared with the control group. Based on this data, it appears reasonable to suggest that the CRKF approach caused less IL-6 inflammatory response in comparison to the response to the fracture itself. Moreover, Sakamoto et al. (18) observed that IL-6 peaked on the first postoperative day and its growth correlated well with the length of operation. We also found a positive correlation between IL-6 concentration and CRKF duration (r=0.587), which is a confirmation of Sakamoto et al. study. It should be noted that our previous study, conducted in peritoneal lavage fluid of children undergoing laparoscopic and open cholecystectomy, also showed a strong, positive correlation between IL-6 and length of operation (19). In the current study, we did not observe IL-8 concentration growth 12-14 hours after the CRKF procedure compared to plasma samples obtained 30 min. before the CRKF procedure, which may be due to the fact that this approach is minimally invasive and without skin incision. This postulate may be also confirmed by our observation which showed that the concentration of IL-8 in plasma of children after the procedure performed was not significantly different than measured in the control group.

According to our knowledge of the available literature, no data is evaluating the involvement of CXCL5 in the inflammatory response to bone fracture. Our study was the first to show that CXCL5 concentration in children with distal forearm fracture 30 min. before the CRKF procedure as well as 12-14 hours after the CRKF procedure did not significantly different compared to the control group. Moreover, we did not observe a statistically significant change of this chemokine concentration 12-14 hours after the CRKF procedure compared to plasma samples obtained 30 min. before the procedure.

In the current study, we also evaluated damage-associated molecular patterns (DAMPs) concentration, as they promote tissue repair and resolution of the inflammation. The release of DAMPs is a highly relevant mechanism by which immune cells can be alerted to tissue damage (20). Taniguchi et al. (21) postulated that HMGB-1, as well as proinflammatory cytokines, are released by damaged cells, suggesting its connection with the process of tissue reorganization.

We showed that HMGB-1 concentration did not change in response to the distal forearm fracture in children. The lack of a significant difference in comparison to the control group may indicate that this kind of injury is not as severe as traumas presented by other authors (22, 23). It may also result from the late response of this DAMP protein to the injury, as postulated by Wang et al. (24). These suggestions are in line with our previous results showing that during open cholecystectomy HMGB-1 concentration is almost the same at the beginning as at the end of the procedure (19).

In contrast to HMGB-1 concentration, we observed significant growth of plasma HSP-70 concentration in children with distal forearm fracture compared to the control group. It may indicate that among DAMPs HSP-70 responds earlier to bone injury in comparison to HMGB-1. It is evidenced that HSPs play important roles in situations involving both systemic and cellular stress (25). Giffard et al. (26) showed that HSP-70 expression was observed after myocardial infarction and cardiac surgery with bypass. Based on their findings it appears reasonable to suggest that increased HSP-70 levels may indicate both tissue damage and response to surgical stress (26). Moreover, induction of HSP expression may effectively reduce cellular injury by accelerating the recovery of damaged cells (27). These results could explain our findings, indicating that HSP-70 concentration was statistically increased in response to distal forearm fracture.

Results concerning the kinetics of HMGB-1 and HSP-70 in the plasma of children after closed reduction with K-wire fixation raise some points of interest. We observed, that concentrations of both DAMPs decreased 12-14 hours after the CRKF procedure. In this context, it is interesting to record that Cohen et al. (23) postulate that in humans HMGB-1 is released to the plasma within 45 min. after severe trauma. Moreover, its release requires severe tissue injury and tissue hypoperfusion, and it is associated with severe inflammatory response and posttraumatic coagulation abnormalities (23). Kim et al. (28) demonstrated that HMGB-1 growth after cardiac surgery reflects extensive systemic inflammation and it is connected with a greater incidence of morbidity and significantly longer duration of postoperative hospitalization (28). There is also evidence that HSP-70 mediates inflammatory response and may be associated with complications after surgery. Kimura et al. (29) observed that plasma HSP-70 concentration was significantly higher in patients with postoperative infection and organ dysfunction after liver resection (29). Based on our present findings, it appears reasonable to postulate that the lowering levels of both DAMPs after surgery in children with distal forearm fractures may indicate that the CRKF procedure is minimally invasive and without risk of postoperative complications and long term hospitalization. This suggestion may also be confirmed by the fact that both HMGB-1 and HSP-70 concentrations 12-14 hours after the CRKF procedure were not significantly different than concentrations of these markers in healthy children. Moreover, the advantage of K-wire fixation is that there is no scarring form surgical dissection, the space for postoperative bleeding is reduced and this type of fixation allows bone to heal in a good position (30), which altogether reduces the extension of inflammatory response.

CRP concentration may reflect the development and augmentation of an inflammatory response, as we showed in several of our previous studies (19, 31–33). In the current study, we noted statistically significant differences in CRP concentration between patients 30 min. before the CRKF procedure as well as 12-14 hours after the CRKF procedure compared to the control group. Comparing CRP concentrations before and after the CRKF procedure, we found that a significant increase of this acute-phase protein was observed 12-14 hours after the performed procedure. However, it should be noted that in all cases the median CRP concentration was within the reference values not indicative of the inflammatory state (12).

Several studies demonstrated that IL-33 is passively released after tissues damage, which may suggest that this alarmin cytokine alerts the immune system after endothelial or epithelial cell damage during infection, physical stress, or trauma (34, 35). These suggestions are generally confirmed by Halat et al. (34), who found that IL-33 levels increased in all patients immediately after they had sustained multiple injuries. Their experiment showed that this alarmin in response to polytrauma indicates structural cell damage (34). In contrast to the above data, our study demonstrated that IL-33 concentrations in all plasma samples of children with distal forearm fracture prior and 12-14 hours after the CRKF procedure were below the detection limit of the assay kit. It may indicate, that this type of fracture is not a severe injury and also confirms that the CRKF approach is not associated with severe tissue damage or extensive inflammatory response.

One of the study limitations could be the fact, that in our children population, only two patients from twenty-nine had a hospitalization time lasting 2 days. Moreover, none of the patients had intraoperative/postoperative complications. Nevertheless, it would be interesting to evaluate how selected inflammatory cytokines (IL-6, IL-8, CXCL5, IL-33), CRP, and damage-associated molecular patterns (HMGB-1, HSP-70) are related to fracture healing, tissue repair, and outcome (e.g. duration of the hospitalization or complications). However, such an experiment requires selecting and collecting a new group of children, having longer than usual time of hospitalization, and with reported intraoperative/postoperative complications. It would be also interesting to evaluate the concentrations of other inflammatory cytokines, including IL-1β and TNF-α. Conducting these analyzes would be impossible with the current experiment because it would require more amount of plasma. This also could be recognized as a study limitation. Another study limitation is a small sample size. Thus it needs to emphasize, that this kind of study should be interpreted carefully. However, data acquired from such experiments could be used to design larger confirmatory analyses to provide final conclusions.

To conclude, increased values of IL-6, CRP, and HSP-70 represented an early inflammatory response to distal forearm fractures classified as SH-II type according to the Salter-Harris classification system. However, the median CRP concentration was within the reference values not indicative of the inflammatory state. The CRKF procedure may be a good solution for the treatment of bone fractures, as damaged associated molecular patterns – HMGB-1 and HSP-70 – did not significantly differ 12-14 hours after the approach was applied as compared to the control group. Moreover, the growth of IL-6 concentration after the CRKF procedure was 1.5-fold to the level before CRKF, while the increase of this marker in response to the distal forearm fracture was 4.3-fold compared with the control group. Based on this data, it appears reasonable to suggest that the CRKF approach caused less damage and inflammatory response in comparison to the response to the fracture. Nevertheless, further research should be conducted to evaluate how the immunomodulation of selected cytokines and DAMPs may have a therapeutic potential to hasten and improve bone healing.

## Data Availability Statement

The datasets generated and analyzed during the current study are not publicly available but all are kept at the Medical University of Bialystok and are available from the corresponding author on reasonable request.

## Ethics Statement

Approval for this study was obtained from the Bioethics Committee of the Medical University of Białystok (No: R-I-002/45/2018). Procedures were following the ethical standards set out in the Declaration of Helsinki put out by World Medical Association. Written informed consent to participate in this study was provided by the participants’ legal guardian/next of kin.

## Author Contributions

MT: conception and design of the study, acquisition of data, analysis and interpretation of data; visualization, drafting of the article. TG: acquisition of data, revising article for its content. MM: acquisition of data, revising article for its content. JK: methodology, revising article for its content. EM: acquisition of data; revising article for its content. MK: acquisition of data; revising the article for its content. OK-L: conception and design of the study, methodology, analysis, and interpretation of data; visualization; resources; drafting of the article. All authors contributed to the article and approved the submitted version.

## Funding

This research did not receive any specific grant from funding agencies in the public, commercial, or not-for-profit sectors.

## Conflict of Interest

The authors declare that the research was conducted in the absence of any commercial or financial relationships that could be construed as a potential conflict of interest.

## Publisher’s Note

All claims expressed in this article are solely those of the authors and do not necessarily represent those of their affiliated organizations, or those of the publisher, the editors and the reviewers. Any product that may be evaluated in this article, or claim that may be made by its manufacturer, is not guaranteed or endorsed by the publisher.
